# The Relationship between Actual Fundamental Motor Skill Proficiency, Perceived Motor Skill Confidence and Competence, and Physical Activity in 8–12-Year-Old Irish Female Youth

**DOI:** 10.3390/sports5040074

**Published:** 2017-09-27

**Authors:** Orlagh Farmer, Sarahjane Belton, Wesley O’Brien

**Affiliations:** 1 School of Education, Sports Studies and Physical Education Department, 2 Lucan Place, Western Road, University College Cork, Cork T12 KX72, Ireland; 111524727@umail.ucc.ie; 2 School of Health and Human Performance, Dublin City University, Dublin D09 W6Y4, Ireland; sarahjane.belton@dcu.ie

**Keywords:** fundamental movement skills, perceived physical self-confidence, physical performance self-concept, physical activity, female youth

## Abstract

This study examines the relationship between actual fundamental motor skill (FMS) proficiency, perceived motor confidence and competence, and physical activity (PA) among female children (*n*= 160; mean age = 10.69 ± 1.40 years). The Test of Gross Motor Development-2nd Edition (TGMD-2) was used to assess seven FMSs (locomotor, object-control, and stability). Motor confidence and competence were assessed using a valid skill-specific scale, and a modified version of the Self-Perception Profile for Children. PA levels were assessed using self-report (PA Questionnaire for Older Children (PAQ-C)) and classified as low, moderate, and high active. One-way and two-way ANOVAs (post-hoc honest significant difference (HSD)) and correlation coefficients were used to analyse the data. Findings indicate that the majority of youth (71.8%) were not meeting the minimum 60 min of daily PA recommended for health, and that 98.1% did not achieve the FMS proficiency expected for their age. While there were high levels of perceived physical self-confidence (PSC) reported within FMS skill-specific tasks, there was no significant correlation observed between actual FMS proficiency and perceived PSC among the cohort. Results show that low, moderately, and highly active female participants differ significantly in terms of their overall FMS (*p* = 0.03) and locomotor (LOC) control scores (*p* = 0.03). Results from a two-way between-groups analysis of variance also revealed no statistically significant interaction effect between PA grouping and physical performance self-concept (PPSC) on overall FMS proficiency levels. Results of a multiple linear regression indicate that perceived PSC is a significant predictor (beta = 0.183) of participants’ overall PA levels. Data show a need for targeting low levels of PA, and low FMS proficiency in female youth, and for developing interventions aiming to enhance perceived PSC levels.

## 1. Introduction

Regular participation in physical activity (PA) and sport is positively associated with an array of physical, psychological, and social health benefits [1]. Despite the widely known positive health benefits of PA, children and adolescents are not meeting the daily recommendations of at least 60 min of moderate to vigorous physical activity (MVPA) [2].

Further to these low levels of PA participation, research consistently indicates a gender-based disparity in PA amongst youth, with girls significantly less active than boys [3]. Research also demonstrates that PA decreases significantly during the transition from childhood to adolescence, with girls in particular showing sharper declines in participation [4]. There is a need to pay attention to girls’ PA patterns and influences [5]. Numerous factors have been shown to influence child PA participation [6], and research suggests that actual movement competency (e.g., fundamental movement skill (FMS) proficiency) in early childhood may be an important prerequisite for engagement in PA later in life [6].

Actual movement competency, which also has been noted in previous literature as motor coordination, motor skill proficiency, FMS, or motor ability [7], can be defined as the basic observable building blocks [8] for movement. These provide the foundation for the specialized, and sport-specific movement skills [9] required for participation in a variety of physical activities including games, sports, and recreational activities. FMS can be categorized as locomotor (e.g., run, skip, jump), object-control (e.g., throw, catch, kick), and stability (e.g., static balance) skills [10]. Children move and engage in PA through the execution of FMS [11]. Previous research has demonstrated positive associations between FMS and areas of health including PA, organized youth sport, and self-concept [12]. Despite FMS contributing to the general development and wellbeing of young children, literature consistently illustrates that proficiency in FMS among children and adolescents is low [13], with only 50% of children demonstrating competency in a broad range of skills [10,14]. By the time children reach 10 years of age, they have developmental capabilities to master FMS performances, [10] however, this is often not the case. These lower levels of FMS proficiency may translate into a lack of confidence in performing specific skills [15]. Research suggests that without FMS proficiency and a positive perception of such, children may be less likely to engage in PA [12]. To better understand the acquisition of FMS alongside levels of PA, it is crucial to consider mediators, such as confidence, that may account for the motor development of children [12]. 

Within the realm of motor development, various terms such as “self-confidence”, “self-efficacy”, “perceived ability”, and “perceived competence” have been used to describe one's perceived capability to accomplish a certain level of performance [16]. Perceived competence refers to an individual’s perception of their actual movement capabilities [17]. Stodden and Goodway [18] postulate that higher perceived competencies are related with FMS proficiency, and increased levels of PA. Similarly, self-confidence refers to the perceived ability to accomplish a certain level of performance [16]. Research carried out in this field suggests that as children gain confidence in performing fine and gross motor skills, and build a sufficiently diverse movement repertoire, they acquire a high level of movement proficiency that is positively associated with the quality of their psychomotor and cognitive health [19]. 

Like self-confidence, research has shown that PA and self-concept are connected in different ways [20]. Physical self-perceptions, including physical performance self-concept (PPSC), are significant correlates of PA in children [21]. Indeed, increasing perceptions of competence and levels of FMS proficiency are potential strategies to promote PA. 

Various studies have been conducted assessing the relationship between perceived movement and actual movement competence [9,22,23] among children, while far fewer refer to perceived confidence (perception of ability/self-efficacy) [23,24], and specifically so within the female childhood population. A recent Irish study investigating the relationship between FMS proficiency and perceived physical self-confidence (PSC) levels among adolescents found a significant correlation between both variables for females [25]. 

Little is known regarding the influences of FMS and perceived movement self-confidence on PA among Irish pre-adolescent girls. At present, there are no studies examining the relationship between perceived PSC, PPSC, and actual FMS involving Irish female children. Consequently, the aim of the present study was to analyse the relationship between perceived PSC, PPSC, actual FMS, and PA in Irish female children.

## 2. Materials and Methods

### 2.1. Participants and Study Design

**Figure 1:**
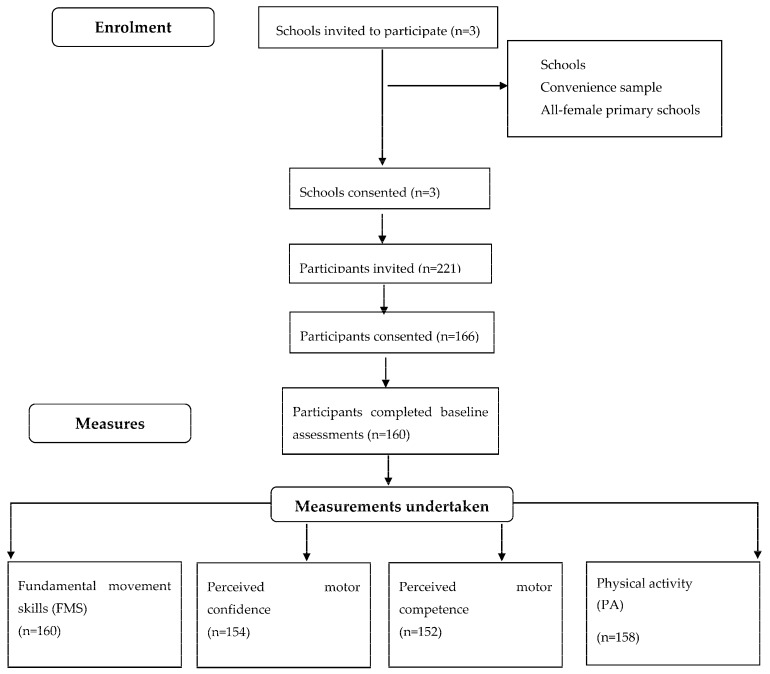
Study design and flow of data collection (*n* = 160).

Two hundred and twenty-one participants from three primary schools were originally invited to participate in the study, with full consent received from 166 participants (75% of total sample). In total, 160 female participants (with a mean age of 10.69 years (SD = ±1.40) had full data available for all measurements, including FMS assessment, the PA questionnaire (PAQ), and the PSC and PPSC scales, as presented in Figure 1.

This cross-sectional, mixed methods study was granted full ethical approval by the Institutional Social Research Ethics Committee at University College Cork (Social Research Ethics Committee, UCC) in 2016. Informed assent for participation was granted by all participants and consent from their parent(s)/guardian(s) and the school principals prior to participation; all participants were free to withdraw from the research at any stage. 

### 2.2. Recruitment and Data Collection 

Convenience sampling was used to recruit three all-female primary schools (rural and urban) in the Cork region, Ireland. A representative social economic status demographic was selected, with the inclusion of one school identified as ‘DEIS’ (delivering equality of opportunity in schools). The lead researcher emailed the principals of all three schools, calling for expressions of interest, and written consent to participate in the data collection process. To be eligible for the study, female participants needed to be formally enrolled in between the years of second to sixth class, and were required to provide written assent and parental/guardian consent prior to participation. If a participant or their parent/guardian did not consent, they were not permitted to participate in the data collection.

Prior to data collection, ten field staff underwent a rigorous and robust 8-hour training workshop (across two days) in the measurement protocol associated with FMS and self-report questionnaires. This involved an objective criteria-informed process to ensure field staff were consistent in the data collection measurement protocol. 

The data were collected on participants within their class groups (max *n* = 30) during specific school visits (*n* = 3). Objective measurements, such as FMS, were carried out during a timetabled block, with a ratio of one researcher to five students. Subjective self-report measurements took place in a supervised classroom, or computer lab, and the ratio of participant to researcher was 10:1. The study was briefly explained and instructions provided on how to complete the questionnaire. Participants were encouraged to take their time, reflect on their answers, and to be as honest as possible. All questionnaires were completed online through the tool ‘Survey Monkey’. In cases where computer networks failed, participants completed hardcopies of the questionnaire. Throughout the duration of this baseline data collection, participants were assigned identification numbers for anonymity purposes. 

#### 2.2.1. PA Self-Report Assessment 

Moderate to vigorous PA (MVPA) was assessed using a modified version of the Physical Activity Questionnaire for Older Children (PAQ-C) [26]. Studies have established the reliability and validity of the 7-day recall on children [27]. The PAQ-C for this study included 15 physical activities, 10 leisure/free-time activities, and activities in school (Physical Education), transport activities (walking to and from school) and other activities. The participants were told to recall what activities they had engaged in the previous seven days and how many times and number of minutes they participated in each of these activities. Habitual PA was also assessed using two questions from the Physician-based Assessment and Counseling for Exercise (PACE) questionnaire: how many days in the last week (PACE 1) and in a usual week (PACE 2) does the subject do at least 60 min of physical activity. The PACE questionnaire presents a test–retest reliability assessed by the Intra-class Correlation Coefficient (ICC) of 0.77 [28], and due to its simplicity and ease of understanding, the PACE questionnaire was suitable for the 8–12-year-old cohort. In the current study, the Cronbach alpha coefficient was 0.63, suggesting that the scale has good internal consistency. Data were collected on participants in their class groups with a ratio of one trained field staff to ten students, for questionnaire completion. The study was briefly explained, and instructions were provided on how to complete the questionnaire. The compound result was obtained from both questions ((PACE 1 + PACE 2)/2) and students were categorized as low active (meeting guidelines on 0, 1, 2, or 3 days a week), moderately active (meeting on 4 or 5 days), or highly active (meeting on 6 or 7 days).

#### 2.2.2. Measures: Fundamental Movement Skills (FMS)

The FMS proficiency of seven movement skills were assessed (*n* = 160) in conjunction with the behavioural components from three established instruments, namely the Test of Gross Motor Development (TGMD) [29], Test of Gross Motor Development-2 (TGMD-2) [30], and the Get Skilled Get Active resource [31]. Each of these instruments and their associated protocols have established validity and reliability in children, and are designed to give an objective measurement of gross motor skill proficiency. The test consists of two subscales, locomotor control (LOC) skills and object-control (OC) skills, and was designed to measure criterion elements of FMS performance in children aged 8–12 years. The seven-item test included: three LOC skills (run, skip, and vertical jump), one stability (balance) skill, and three OC skills (stationary dribble, catch, kick), which combine to give an overall maximum raw score of 60. A total score for all seven skills was calculated for each participant, along with an OC score, and LOC score. Overall mean OC and LOC scores were also calculated. The raw skill scores were then added to obtain a raw LOC subtest score (ranging from 0–36 points) and a raw OC subtest score (ranging from 0–24 points). 

During data collection, the seven skills were assessed during a three-hour timetabled block and one trained field staff member provided every participant (in groups of 8) with an accurate demonstration of the FMS to be performed. Participants performed the skill on three occasions, including one familiarization practice, and two performance trials, as reported in previous Irish adolescent movement skill data collection studies [13,32]. Participant performance, along with execution of the required skill, were recorded using digital video cameras (3× Canon type Legria FS21 cameras; Canon Inc., Tokyo, Japan) to allow for greater measurement scrutiny, and accuracy of measurement precision during analysis. The FMS scoring process was completed at a later date by the principal investigators. The number of FMS performance criteria varied from six to twelve across the range of selected FMS. There were a total of 29 performance criteria for all seven fundamental movement skills. Once data collection was completed, the principal investigators were required to reach a minimum of 95% inter-observer agreement for scoring all seven FMS. If children displayed correct performance on all or all but one skill component, they were classified as having achieved ‘mastery’ or ‘near mastery’, respectively, for that skill. ‘Near mastery’ was defined as correct performance of all components but one on both trials [33].

#### 2.2.3. Perceived Physical Self-Confidence

Participants’ perceived PSC levels were assessed using the PSC scale [34], which has shown excellent test–retest reliability, and internal consistency, with a Cronbach alpha coefficient of 0.94 [34]. In the current study, the Cronbach alpha was 0.88, suggesting very good internal consistency reliability for the perceived PSC scale for the seven skills with this sample. The PSC scale consists of 15 questions in which participants rate their confidence at performing 15 separate FMSs. The identified skills included within this instrument are considered central to the Irish youth sporting culture [8,33]. Participants rated their confidence at performing each skill on a Likert scale of 1–10, with “1” being not confident at all, and “10” being very confident. This present study assessed 7 of the 15 actual movement skills, consistent with the seven actual FMSs assessed, therefore, the maximum PSC score which could be achieved was 70 if participants scored their confidence at 10/10 for performing all seven skills. Similar to previous research [25], participants were divided into three tertiles based on perceived PSC using visual binning in SPSS (≤28.7 was the low PSC group, 28.8–49.3 was the medium PSC group, and 49.4+ was the high PSC group).

#### 2.2.4. Physical Performance Self-Concept

Participants’ PPSC were assessed using a modified version of the Athletic Competence sub-scale, taken from the Self-Perception Profile for Children [35,36]. The scale for this study consists of eleven statements (e.g., ‘I am good enough at sports’). Students were asked to indicate how true each statement was for them (‘very true’, to ‘not at all true’, range 1–4), and the participant selected the statement that best described them. The more positive the statement in the questionnaire, the higher the value. For example, 1 = not at all true and 4 = very true for nine of the ‘positive’ statements (e.g., ‘I feel positive about myself physically’ and ‘I am good enough at sports’, etc. 1 = not at all true and 4 = very true). This was reversed and recoded for two of the ‘negative’ PPSC statements (i.e., ‘I wish I could feel better about myself physically’ and ‘I like to watch sports rather than play’), whereby in this case 4 = not at all true and 1 = very true. Reliabilities for the subscales of the PPSC range from 0.73 to 0.84 [35], and the test-retest reliability coefficient (r = 0.51) was moderate [36]. In the current study, the Cronbach alpha coefficient was 0.70, thus suggesting that the scale has acceptable internal consistency.

### 2.3. Data Analysis 

The FMS, PSC, PPSC, and PA data set were analysed using IBM, USA for SPSS software (version 20.0 for Windows (SPSS Inc., IBM Corp., Armonk, NY, USA). Statistical significance was set at *p* < 0.05. Where participants had incomplete data for a given variable, participants were excluded from analysis of this variable specifically. The number of days participants self-reported meeting the 60 min PA guidelines were analysed descriptively using means, standard deviations, and proportions. Descriptive statistics and frequencies for all variables were calculated. 

One-way between-groups analysis of variance were conducted to explore the impact of PA groupings (low, moderately, and highly active) on overall FMS, LOC and OC proficiency levels, perceived PSC and PPSC levels. As the assumptions for ANOVA were met, two-way between-groups ANOVAs were used to explore the impact of the two variables (perceived PSC, PPSC) and PA grouping (low, moderately, or highly active) on total FMS, LOC, and OC scores. In instances where significant main effects were found, post-hoc comparisons were carried out using the Tukey (HSD) test to determine where the differences occurred. Considering the relatively large numbers of means (FMS, LOC, and OC), the use of Tukey HSD comparisons are a powerful, and accepted form of statistical post-hoc assessment.

Spearman’s correlation coefficient test was used to investigate the relationship between actual FMS proficiency levels and perceived PSC levels. A multiple linear regression was conducted to explore the predictive ability of actual FMS, perceived PSC, and PPSC on PA measurement. 

## 3. Results

### 3.1. Physical Activity

Self-report PA data showed that 10.2% of participants were meeting the 60 min guideline on 0–3 days a week (low active), with 40.95% meeting the guideline on 4 or 5 days a week (moderately active), and the remaining 48.85% of participants meeting the guidelines on 6 or 7 days a week (highly active). The percentage of participants meeting the 60 min PA guideline on all 7 days was 28.2%. 

#### 3.1.1. Fundamental Movement Skills

Only three participants (1.9%) possessed complete mastery level across all seven OC and LOC skills. Overall, the vertical jump and the skip were the poorest performed skills across the cohort, where 38.6% and 36.9%, respectively, achieved mastery. The best performance was for the kick (OC), where 68.4% and 24.7%, respectively, achieved mastery and near-mastery. The overall mean FMS score of participants was 48.75 (SD = ±5.83), out of a possible score of 62. Mean overall FMS scores for the three PA groups were as follows: low = 46.25 (SD = ±7.60), medium = 49.92 (SD = ±3.80), and high = 48.74 (SD = ±6.04). Mean skill scores by PA grouping (low, moderately, and highly active) for actual FMS are given in Table 1. A one-way between-groups analysis of variance was conducted to explore the impact of PA groupings (low, moderately, and highly active) on overall FMS proficiency levels. Participants were divided into three groups according to their PA level (Group 1: low active; Group 2: moderately active; Group 3: highly active). There was no statistically significant difference at the *p* < 0.05 level in overall actual FMS proficiency for the three PA groupings.

#### 3.1.2. Perceived Physical Self-Confidence

Interestingly, 40 participants (26% of the cohort) rated themselves as the top score on the perceived PSC scale for all seven FMSs, while only three participants (1.9% of the cohort) possessed complete mastery level across all seven FMSs in the actual FMS assessment. The overall mean perceived PSC score of participants was 57.64 (SD = ±14.23), out of a possible score of 70. Mean skill scores by PA grouping (low, moderately, and highly active) for perceived PSC are given in Table 1. There was also a statistically significant difference at the *p* < 0.05 level in overall perceived PSC scores for the three PA groups (F (2, 143) = 3.34, *p* = 0.038). There were significant PA grouping differences observed in perceived PSC, with highly active participants scoring significantly higher than low active participants in two of the seven individual skills, as highlighted in Table 1. This included one LOC skill (run; *p* = 0.020) and one object control skill (catch; *p* = 0.046). 

#### 3.1.3. Physical Performance Self-Concept

The overall mean PPSC score of participants was 18.89 (SD = ±3.31), out of a possible score of 24. Mean skill scores by PA grouping (low, moderately, and highly active) for PPSC are given in Table 1. A one-way between-groups analysis of variance was conducted to explore the impact of PA grouping on overall PPSC levels. There was a statistically significant difference at the *p* < 0.05 level in PPSC scores for the three PA groups (F(2, 147) = 7.32, *p* = 0.001). The actual difference in mean scores between the groups was moderate. The effect size, calculated using eta squared, was 0.09, which in Cohen’s [37] terms would be considered a medium effect size. Post-hoc comparisons using the Tukey HSD test indicated that the mean score for highly active (M = 19.54, SD = 2.89) participants was significantly different (*p* = 0.05) from the low active participants (M = 16.64, SD = 3.63). Likewise, the mean score for the moderately active group (M = 17.89, SD = 3.80) was significantly different than the highly active group (*p* = 0.02).

### 3.2. Interrealtionship between Variables

A two-way between-groups analysis of variance was conducted to explore the impact of PA groupings and perceived PSC on overall FMS proficiency levels. Participants were divided into three groups according to their PA level (Group 1: low active; Group 2: moderately active; Group 3: highly active). The interaction effect between PA grouping and perceived PSC was statistically significant (F(4, 143) = 2.76, *p* = 0.03). There was also a statistically significant main effect for PA category (F(4, 143) = 4.76, *p* = 0.01). 

Additionally, a two-way between-groups analysis of variance was conducted to explore the impact of PA grouping and perceived PSC on overall FMS LOC proficiency levels, as shown in Table 2. The interaction effect between the two variables was statistically significant (F(4, 143 = 2.78, *p* = 0.029). There was also a statistically significant main effect for PA category (F(2, 143) = 6.54, *p* = 0.002), and PSC grouping (F(2, 143) = 3.77, *p* = 0.025). This means that low, modertely, and highly active participants differ in terms of their overall locomotor scores, and there is also a difference in scores for participants with low, modertely, and highly perceived PSC scores. Results from a two-way between-groups analysis of variance also revealed no statistically significant interaction effect between PA grouping and PPSC on overall FMS profocency levels. 

The relationship between actual FMS proficiency levels and perceived PSC levels was investigated using Spearman’s correlation coefficient. Preliminary analyses were performed to ensure no violation of the assumption of normality, linearity, and homoscedasticity. There was no correlation found between the two variables (rho = 0.11, *n* = 154, *p* = 0.191).

A multiple linear regression was calculated to predict overall PA levels based on participants’ perceived PSC, PPSC, and actual FMS proficiency. A significant regression equation was found (F(3, 145) = 3.127, *p* = 0.028), with an R^2^ of 0.061. The model explains only 6.1% of the variance in overall PA levels. Of the three variables, perceived PSC makes the largest, unique, significant contribution (beta = 0.183) while PPSC and actual FMS proficiency made no statistical contribution (beta = 0.119 and 0.011, respectively). This indicates that PSC is a predicting variable in female participants’ overall PA levels.

## 4. Discussion

Results of this study show that the percentage of participants meeting the 60 min MVPA guidelines on all 7 days was 28.2%. These low levels of PA participation are relatively consistent with other studies on a national Irish level [38,39]. Although higher than the 16% reported figure for a similar age group in the Irish Health Behaviour in School-aged Children (HBSC) study [40], these low levels are in line with this study’s findings. 

The majority of female adolescents in this study (98.1%) failed to reach a level of mastery across key FMSs, indicating that basic movement skill proficiency amongst this selected pre-adolescent Irish female youth cohort is low. These observed low findings of FMS proficiency are consistent with recent Irish adolescent research [13]. Results from the present cohort indicate that only three participants were fundamentally competent across all seven object-related, stability, and locomotor skills, despite children having the developmental capacity to master these skills by ten years of age [10]. Previous international research examining the FMS proficiency of female children support these low levels of FMS competency [41,42].

In the present study, 26% of participants (*n* = 40) rated themselves as being fully proficient at performing the seven FMSs on the perceived PSC scale. However, actual FMS assessment results reveal only three participants (1.9%) possessed complete proficiency across all seven FMSs. On the perceived PSC scale (0–10), participant mean values were generally in the upper thresholds (mean values of ≥6.5 within Table 1), indicating higher levels of perceived PSC amongst this selected female cohort. High reported PSC in the current study aligns with recent research on an Irish cohort, which highlighted that female adolescents in particular consistently scored a mean of 6.8 or above (out of 10) in confidence, regardless of their actual ability [25]. Similarly, in another recent Irish adolescent study [43], the results purport that participants had considerably higher perceived PSC levels when compared to their actual skill proficiency in FMS. 

Futhermore, the results of this study suggest that despite the low levels of actual FMS proficiency, Irish girls may be inaccurately overestimating their perceived confidence levels for movement. Harter [44] supports this view in postulating that it is considered ‘normative’ for young children to overestimate their abilities, because of cognitive limitations in finding it hard to distinguish between their ‘ideal’ in terms of competence, and their own reality [44]. Research suggests that inflated perceived competence can drive the acquisition of movement skills, because children will continue to engage in mastery attempts in activities for which they believe they are skilful [18]. Furthermore, this early period has been termed a ‘window of opportunity’, as children (even if low skilled) can still be keen participators in activity [45]. It is, therefore, imperative to have an understanding of children’s perceived and actual movement skill proficiency to allow for the implementation of interventions during this early period. 

In this study, no correlation was found between perceived PSC and actual FMS proficiency levels. This indicates that children’s perceptions of their LOM and OC skill proficiencies are not associated with the ‘reality’ in terms of their actual FMS proficiencies. This lack of association in the current study can perhaps be explained by the fact that the participants’ mean age was 10.69 years (SD = ±1.40), and according to Barnett et al. [46], young children may not have the capacity to differentiate perceived competence in terms of the different skill types of LOM and OC. The findings in the current study are not in line with McGrane et al. [25], where perceived PSC and actual FMS proficiency levels were moderately correlated among Irish females (r = 0.305). These findings [25] suggest that if a female has low actual FMS proficiency, she may in turn have low PSC levels, or vice versa. Likewise, in a recent study by O’ Brien et al. [43], the results revealed that the perception of adolescent females in relation to their movement confidence did not equate to their actual movement skill proficiencies. Similar results were also found in the Vedul-Kjelsås et al. [47] study, where actual FMS proficiency and self-perception were strongly correlated with girls (r = 0.312, mean age 11.46 years). Nevertheless, the results of the current study align with Stodden et al.’s [18] developmental theory, which suggess that the transition into middle childhood (9–11 years) marks an important period when perceived physical competence should be lower. Stodden et al. [18] postulate that the shift from early childhood to middle childhood marks the beginning of a period of vulnerability during which children who have lower actual movement skill competence will demonstrate lower perceived movement skill competence and are less physically active.

Consistent with other studies [48,49], the analysis of psychological variables reveals an association with female children’s PA levels. Although there were no associations found in the current study, there were significant differences found among the PA groupings (low, moderately, and highly active) for perceived PSC and LOM scores and overall FMS levels, which exhibited small effects. The interaction effect between PA grouping and perceived PSC was statistically significant. There was also a statistically significant main effect for PA category. This indicates that there is a significant difference in the effect of perceived PSC on overall FMS scores for the different PA groupings (low active, moderately active, and highly active). Furthermore, this implies that PA groupings may impact perceived PSC levels. Research suggests that those who are not confident about their ability (in this case those in the low perceived PSC group) will not want to put themselves in a situation where they may display low ability levels, which in turn may affect their performance [44]. Furthermore, a comprehensive understanding of the issues surrounding perceived PSC and motivation for PA by developmental stages in female adolescents are vital for improving interventions, and enhancing psychological well-being [50]. 

To the authors’ knowledge, this is the first study of its kind in Ireland that seeks to critically examine late childhood, and early adolescent female perceived PSC, PPSC, actual FMS proficiency, and PA participatory levels. Nevertheless, one limitation of this current study is the use of a convenience sample, which can lead to the under-representation or over-representation of particular groups within the sample. Another limitation of the present study is its cross-sectional design. The results do not provide causal evidence regarding relationships among actual FMS proficiency, perceived PSC, PPSC, and PA. To gain more insight in the direction of these relationships and to understand how associations among these variables may change over time, longitudinal or experimental studies should be conducted. 

## 5. Future Practical Implications

Pre-adolescent girls are an important target for PA behavioural change strategies, as this age cohort may enhance tracking into the crucial period of adolescence [51]. The quantification of intervention effectiveness for this age group of girls has not been adequately reported [51] specifically examining perceived PSC and actual FMS proficiency. Indeed, as investigated in the present cross-sectional study, components that foster the development of both actual and perceived confidence levels may have the potential to significantly improve the long-term impact of childhood and adolescent movement. 

Considering the observed low levels of actual FMS proficiency amongst female pre-adolescents, developing a specifically designed movement-oriented intervention would be a strategic step towards improvement. Furthermore, the promotion of autonomous and competence-based activities among PA groupings (particularly targeting low active girls) may exist within future interventions. From this study, it appears that females need additional hours of instructional practice towards the acquisition of actual FMS proficiency.

## 6. Conclusions

In terms of perceived PSC, participants generally displayed higher levels of confidence, however, these results do not appear to be associated with their actual movement-based tasks. Results from this study suggest that future interventions may need to specifically address the low levels of actual movement skill proficiency with developmentally appropriate strategies for understanding perceived confidence at the FMS level. Likewise, increasing perceptions of PSC and levels of actual FMS proficiency are potential strategies to promote physical activity [52]. Furthermore, physical education professionals and youth sports programmes should target both actual and perceived motor confidence and competence in order to promote lifelong PA in children.

## Figures and Tables

**Table 1:** Descriptive data for each variable (frequency, means, standard deviation, and physical activity (PA) grouping (low, moderately, and highly active).

Frequencies of PA Groupings						
Variable	N	M	SD	LA	MA	HA
**PA**	158	1.57	0.67	16	36	106
**FMS**	158	48.75	5.84	16	36	106
**PSC**	152	57.55	14.34	15	35	102
**PPSC**	150	18.89	3.33	14	35	101
**LOC**	158	29.71	4.20	16	36	106
**OC**	158	19.04	2.98	16	36	106
**Age**	160	10.69	1.40	16	36	106

N = number; M: Mean; SD: standard deviation; LA: low active; MA: moderately active; HA: highly active; LOC: locomotor control; OC: object-control.

**Table 2:** Mean scores for fundamental movement skills (FMSs), perceived physical self-confidence (PSC), and physical performance self-concept (PPSC) by physical activity (PA) grouping (low, moderately, and highly active).

	FMS (*n* = 158)				PSC (*n* = 152)			
Skill	Low Act (*n* = 16)	Mod Act (*n* = 36)	High Act (*n* = 106)	OMS	Low Act (*n* = 15)	Mod Act (*n* = 35)	High Act (*n* = 102)	OMS
Run	6.79 SD = ±1.42	6.67 SD = ±1.51	6.59 SD = ±1.41	6.63 SD = ±1.43	6.53 SD = ±3.50 *	7.06 SD = ±3.12	8.27 SD = ±2.66 *	7.81 SD = ±2.92
Skip	4.60 SD = ±1.18	5.00 SD = ±1.33	4.63 SD = ±1.12	4.72 SD = ±1.18	6.93 SD = ±3.22	6.86 SD = ±3.27	7.90 SD = ±2.71	7.56 SD = ±2.92
VJ	9.67 SD = ±2.38	9.94 SD = ±2.01	9.97 SD = ±2.24	9.94 SD = ±2.19	7.13 SD = ±3.25	7.26 SD = ±3.23	8.19 SD = ±2.72	7.87 SD = ±2.92
Balance	8.63 SD = ±1.31	9.17 SD = ±1.16	8.73 SD = ±1.34	8.82 SD = ±1.30	8.27 SD = ±2.34	8.15 SD = ±2.55	8.99 SD = ±2.07	8.73 SD = ±2.23
Kick	7.13 SD = ±1.50	7.58 SD = ±0.77	7.37 SD = ±1.04	7.39 SD = ±1.05	8.33 SD = ±1.92	8.09 SD = ±2.78	8.88 SD = ±1.88	8.65 SD = ±2.13
Bounce	6.06 SD = ±2.21	6.60 SD = ±1.99	6.77 SD = ±1.68	6.66 SD = ±1.81	9.27 SD = ±1.44	8.15 SD = ±2.85	9.08 SD = ±2.10	8.89 SD = ±2.26
Catch	5.13 SD = ±1.20	5.11 SD = ±1.01	5.43 SD = ±0.92	5.33 SD = ±0.98	7.07 SD = ±2.99 *	8.62 SD = ±2.26	8.72 SD = ±2.34 *	8.53 SD = ±2.43
LOC	27.94 SD = ±6.04	30.81 SD = ±2.71	29.60 SD = ±4.22	29.71 SD = ±4.20	7.22 SD = ±3.08	7.33 SD = ±3.04	8.34 SD = ±2.54	7.63 SD = ±2.89
OC	18.31 SD = ±3.44	19.11 SD = ±2.46	19.13 SD = ±2.98	19.04 SD = ±2.91	8.22 SD = ±2.12	8.29 SD = ±2.63	8.89 SD = ±2.11	8.47 SD = ±2.29
OMS	46.25 SD = ±7.60	49.92 SD = ±3.80	48.74 SD = ±6.04	48.75 SD = ±5.84	53.53 SD = ±14.48	53.23 SD = ±17.46	59.62 SD = ±12.76	57.64 SD =±14.23 *
PPSC	16.64 SD = ±3.63 *	17.89 SD = ±3.80 *	19.54 SD = ±2.89 *	18.89 SD = ±3.33 *				

*: Statistical significance *p* ≤ 0.05. OMS: Overall mean score; Mod: Moderately; VJ: Vertical jump; LOC: Mean locomotor score; OC: Mean object-control score.
